# Marine- and Plant-Based Nanoemulsion Platforms Enhance the Anticancer Activity of Curcumin In Vitro

**DOI:** 10.3390/ijms27010029

**Published:** 2025-12-19

**Authors:** Mahmoud Hasan, Kamil Elkhoury, Cyril J. F. Kahn, Michel Linder, Elmira Arab-Tehrany

**Affiliations:** 1 LIBio, Université de Lorraine, F-54000 Nancy, France; mahmoud.hasan@univ-lorraine.fr (M.H.); cyril.kahn@univ-lorraine.fr (C.J.F.K.); michel.linder@univ-lorraine.fr (M.L.); 2 Department of Chemical and Biological Engineering, American University of Sharjah, Sharjah P.O. Box 26666, United Arab Emirates

**Keywords:** emulsions, breast cancer, MCF-7 cells, drug delivery, curcumin, in vitro

## Abstract

Curcumin is a natural bioactive compound with demonstrated anticancer activity. However, its poor aqueous solubility and limited bioavailability constrain its therapeutic utility. This study formulated nanoemulsions using marine (salmon oil) and plant (rapeseed oil) lipids to enhance the solubility and delivery of curcumin. The fatty acid profiles and lipid class distributions of both lipid sources were characterized. The resulting nanoemulsions prepared from salmon and rapeseed oils exhibited mean droplet diameters of approximately 170 nm and 220 nm, respectively, and remained physically stable for 30 days at 25 °C. Notably, curcumin-loaded nanoemulsions displayed smaller droplet sizes than their unloaded counterparts, suggesting strong curcumin–lecithin interactions. In vitro cytotoxicity assays demonstrated that the curcumin-loaded nanoemulsions significantly reduced the proliferation of MCF-7 human breast cancer cells (*p* < 0.001). Collectively, these findings indicate that lipid-based nanoemulsions represent a promising delivery platform for curcumin in the context of breast cancer therapy.

## 1. Introduction

Oil-in-water (O/W) nanoemulsions are widely utilized across the food, pharmaceutical, and cosmetic industries for the delivery of hydrophobic bioactive compounds, including vitamins, omega-3 fatty acids, antimicrobials, antioxidants, phytosterols, and parenteral nutrition components [1,2]. These nanoemulsions are typically formulated by homogenizing an oil phase into an aqueous phase in the presence of water-soluble emulsifiers and stabilizers [3]. The distinctive characteristics of nanoemulsions, including small droplet size, surface charge, and large surface area, enable controlled release kinetics and confer excellent stability against coalescence, flocculation, and gravitational separation [4]. Nevertheless, the primary destabilization mechanism in these systems is Ostwald ripening, which occurs due to the diffusion of the dispersed phase through the continuous aqueous phase. This limitation can be mitigated by incorporating dispersed phases with very low aqueous solubility [5].

Nanoemulsion preparation can be accomplished through either low-energy (chemical-based) or high-energy (mechanical-based) approaches [6]. Mechanical methods include ultrasound homogenization, high-pressure homogenization, and microfluidization. Surfactants play a critical role during emulsion formation by reducing interfacial tension between the oil and aqueous phases. Recently, phospholipids have gained attention for formulating pharmaceutically acceptable emulsions [7,8]. Lecithin, characterized by its amphiphilic structure comprising two hydrophobic fatty acid tails, is a major component of cellular lipid bilayers. Its biocompatibility and biodegradable nature position lecithin as an ideal biological surfactant [9].

Marine oils represent a readily accessible source of polyunsaturated fatty acids (PUFAs), which are essential for human health [10]. The omega-3 fatty acids eicosapentaenoic acid (EPA) and docosahexaenoic acid (DHA) are particularly significant in preventing various pathological conditions, including diabetes, inflammation, allergies, hypertriglyceridemia, and coronary heart disease [11]. Notably, long-chain polyunsaturated fatty acids (LC-PUFAs) comprise approximately 35% of total fatty acids in brain tissue [12]. Lipids derived from salmon heads (*Salmo salar*), including both oil and lecithin, exhibit high concentrations of PUFAs, particularly EPA and DHA [13,14]. In contrast, rapeseed lipids consist predominantly of three mono- and polyunsaturated fatty acids: oleic acid (C18:1), linoleic acid (C18:2), and α-linolenic acid (C18:3). Linoleic and α-linolenic acids are classified as essential fatty acids due to the inability of humans to synthesize them endogenously, making them crucial for human health [15,16,17].

Curcumin is a highly lipophilic bioactive compound extracted from turmeric rhizomes (*Curcuma longa*) that exhibits diverse health-promoting properties, including antioxidant, anti-inflammatory, anticarcinogenic, and antimicrobial activities [18,19]. Most research investigating curcumin employs mixtures containing at least three major curcuminoids [20]. However, the compound undergoes rapid metabolism in both intestinal and hepatic tissues, complicating efforts to trace curcumin during absorption, circulation, or excretion [21]. Furthermore, its limited aqueous solubility and poor oxidative stability present significant challenges for incorporating curcumin into pharmaceutical and food formulations [22]. Consequently, there is substantial interest in developing novel encapsulation strategies to enhance curcumin solubility and bioavailability.

In this study, two nanoemulsion formulations based on salmon and rapeseed lipid sources were prepared using a combination of sonication and high-pressure homogenization techniques. These systems were characterized and evaluated for their capacity to deliver hydrophobic curcumin in vitro. The investigation employed comprehensive physicochemical characterization coupled with real-time cell impedance monitoring to assess MCF-7 breast cancer cell responses to curcumin-loaded nanoemulsions.

## 2. Results and Discussion

**Figure 1:**
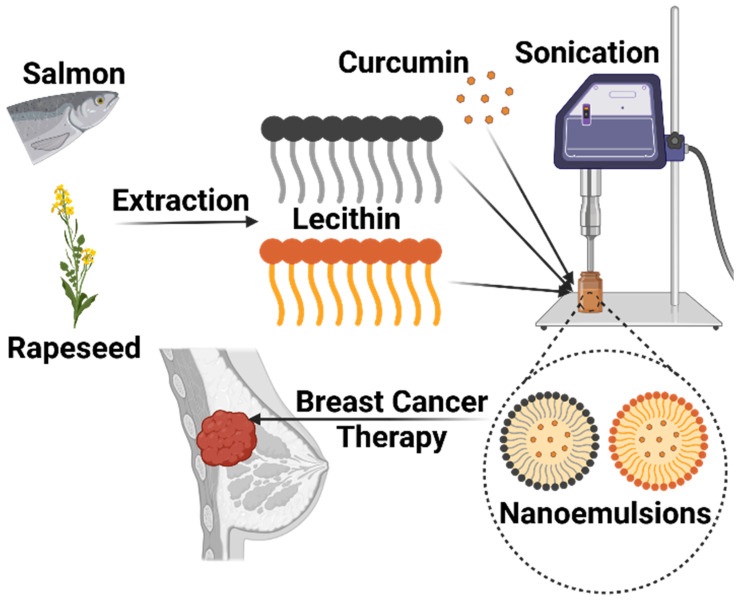
Schematic representation of the breast cancer treatment potential of curcumin-loaded nanoemulsions produced through sonication from lecithin extracted from salmon and rapeseed. Created in BioRender. Kahn, E. (2025) https://BioRender.com/rdh0q8q.

To clarify the conceptual framework and experimental design of this study, Figure 1 provides a schematic outline of the nanoemulsion fabrication process and its intended therapeutic application. This diagram presents the extraction of lecithin from distinct marine (salmon) and plant (rapeseed) sources, followed by the sonication-assisted encapsulation of curcumin. Furthermore, it illustrates the subsequent evaluation of these bioactive nanocarriers for their antiproliferative efficacy against breast cancer cells.

### 2.1. Lipid Classes

**Figure 2:**
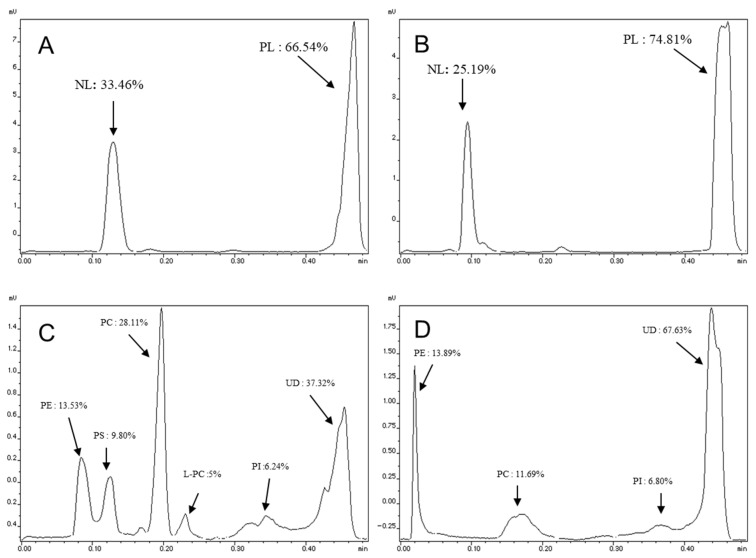
Lipid classes and fractions of polar lipids of salmon and rapeseed lecithin. (**A**,**B**) Lipid classes of salmon and rapeseed lecithin, respectively. (**C**,**D**) polar lipids of salmon and rapeseed lecithin, respectively. PE: Phosphatidylethanolamine, PS: Phosphatidylserine, PC: Phosphatidylcholine, L-PC: Lysophosphatidylcholine, PI: Phosphatidylinositol and UD: Undetermined fraction; (*n* = 10).

The percentages of neutral lipids, polar lipids, and specific lipid classes were quantified using thin-layer chromatography coupled with flame ionization detection (TLC-FID) and are presented in Figure 2. The chromatographic migration of salmon and rapeseed oils revealed the presence of a single peak representing the triacylglycerol (TAG) fraction (100%). These results indicate that the oils used in this study were pure and did not contain any detectable polar fractions.

For the lecithin samples, migration using the apolar solvent system (hexane/diethyl ether/formic acid, 80:20:0.2, *v*/*v*/*v*) revealed the presence of two distinct peaks for both lecithin types (salmon and rapeseed). These two peaks correspond to the polar lipid (PL) and neutral lipid (NL) fractions. The polar and neutral fractions constituted 66.54% and 33.46% for salmon lecithin, respectively (Figure 2A), while the percentage of polar lipids was higher in rapeseed lecithin (74.81%) (Figure 2B).

A second migration using a polar solvent system (chloroform/methanol/ammonia, 65:35:5, *v*/*v*/*v*) allowed for the separation of the different classes of polar lipids. The results of this separation showed that salmon lecithin contains phosphatidylcholine (PC) as the major phospholipid class (28.11%), followed by phosphatidylethanolamine (PE) (13.53%) (Figure 2C). In rapeseed lecithin, the proportion of PE was relatively higher (13.89%) than that of PC (11.69%) (Figure 2D).

### 2.2. Fatty Acid Composition of Oils and Lecithins

The main fatty acid compositions of the lipid sources are shown in Table 1. Analysis of the lipid classes revealed that monounsaturated fatty acids (MUFAs) were the predominant fraction in both oils and rapeseed lecithin. In contrast, salmon lecithin was characterized by a predominance of PUFAs (49.13%).

Specifically, MUFAs represented the highest percentage in rapeseed samples, followed by PUFAs. However, salmon lecithin exhibited a distinct profile where PUFAs constituted the major fraction. Oleic acid (C18:1n-9) was the primary MUFA across all samples, reaching particularly high levels in rapeseed oil (63.02 ± 0.06%) and rapeseed lecithin (53.75 ± 0.23%).

In rapeseed lipids, linoleic acid (C18:2n-6) was the most abundant PUFA (11.46 ± 0.18% for oil and 27.95 ± 0.06% for lecithin), followed by α-linolenic acid (C18:3n-3) (9.22 ± 0.03% and 6.54 ± 0.09% for oil and lecithin, respectively).

For salmon lipids, a significant disparity was observed in the LC-PUFA content between the lecithin and oil, particularly regarding EPA and DHA levels. Salmon lecithin contained approximately twice as much EPA (9.40 ± 0.06%) as salmon oil (4.94 ± 0.02%) and nearly three times more DHA (23.41 ± 0.29%) compared to the oil (6.43 ± 0.04%). Consequently, due to this elevated content of EPA and DHA, salmon lipids exhibited a significantly higher n-3/n-6 ratio compared to rapeseed lipids.

### 2.3. Solubility Test of Curcumin in Oils

Curcumin exhibits extremely low water solubility (<0.005 wt.%) and a moderate oil-water partition coefficient (logP = 2.5) [23]. Consequently, the loading capacity of an emulsion-based O/W delivery system is primarily limited by the maximum concentration of curcumin that can be incorporated into the lipid phase. Accordingly, curcumin must be fully dissolved within the lipid phase prior to emulsification. Solubilization within the oil matrix improves the transport of lipophilic molecules to the intestinal lymphatic system by enhancing absorption from the gastrointestinal tract [24]. The maximum solubility of curcumin in rapeseed oil was determined to be 0.18 ± 0.05 mg/mL, nearly two-fold lower than that in salmon oil (0.33 ± 0.07 mg/mL). This significant difference (*p* < 0.039) can be attributed to the distinct fatty acid compositions of the two lipid sources. While rapeseed oil is predominantly composed of monounsaturated fatty acids (mainly oleic acid, ~63%), salmon oil is characterized by a high content of LC-PUFAs, specifically EPA and DHA.

The higher degree of unsaturation in salmon oil enhances the solvent capacity of the lipid regarding curcumin. The abundance of double bonds in PUFAs increases the polarizability of the lipid chains, thereby facilitating stronger polar interactions with the phenolic and diketone moieties of the curcumin molecule.

This mechanism aligns with partition coefficient modeling, which suggests that lipids with higher polarity and unsaturation levels offer a more thermodynamically favorable environment for solubilizing hydrophobic compounds containing polar moieties, like curcumin, compared to saturated or monounsaturated lipid matrices [23].

### 2.4. Physicochemical and Morphological Properties of Nanoemulsions

**Figure 3:**
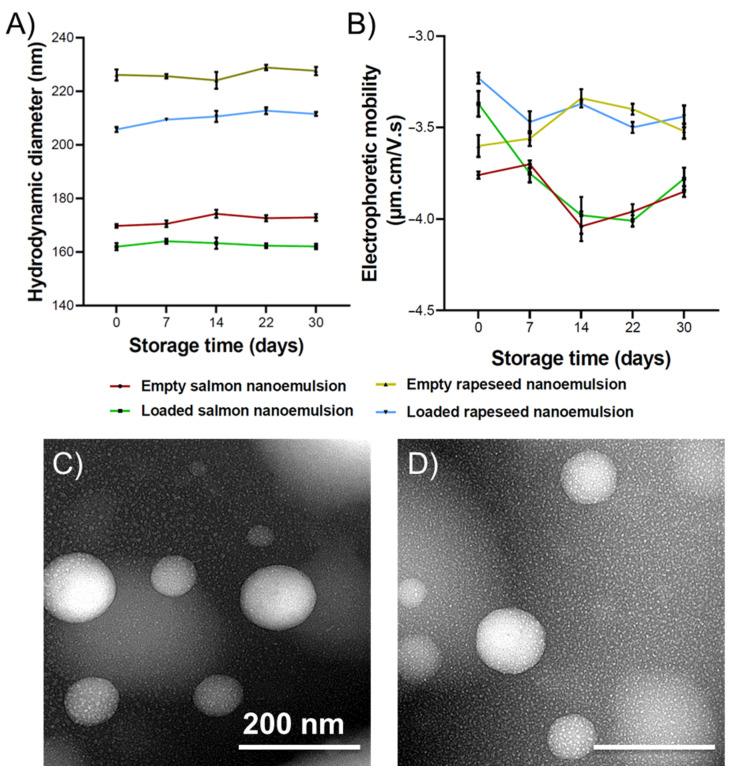
(**A**) Average hydrodynamic diameter and (**B**) electrophoretic mobility of salmon and rapeseed nanoemulsions with and without curcumin, immediately after preparation (T0) and for 30 days stored at 25 °C. The polydispersity index for all formulations during the 30 days was between 0.13 and 0.19; (*n* = 3). TEM image of O/W nanoemulsion composed of (**C**) salmon and (**D**) rapeseed oils and lecithin as a surfactant.

The mean size and size distribution are generally affected by the preparation method and the lipid composition. As shown in Figure 3A, significant differences in mean droplet diameters were observed between the two lipid formulations, as well as between empty and curcumin-loaded systems. The disparity in size between the two nanoemulsion types is likely attributed to differences in fatty acid composition and the lipid class distribution of the lecithins. Specifically, salmon lecithin contains a significantly higher proportion of phosphatidylcholine (PC) (28.11%) compared to rapeseed lecithin (11.69%) (*p* < 0.001), while phosphatidylethanolamine (PE) levels remain comparable. This demonstrates that the size of nano-droplets depends not only upon physical processing parameters but also on the oil composition and the surface-active properties of the lipids employed [25,26,27]. Furthermore, the high content of PUFAs in salmon lipids likely influences the molecular packing within the droplet core [28].

Notably, the reduced size observed in curcumin-loaded nanoemulsions is likely due to strong hydrophobic interactions between curcumin and the lipid matrix, resulting in a more compact core structure. These findings align with previously reported data [25,29].

Electrophoretic mobility values (Figure 3B) were higher in magnitude for rapeseed nanoemulsions compared to salmon and for empty systems compared to their loaded counterparts. A higher magnitude of electrophoretic mobility generally indicates greater electrostatic repulsion, which prevents particle aggregation. The negative surface charge of these nanoemulsions is attributed to anionic phospholipids, such as phosphatidylinositol (PI), phosphatidylglycerol (PG), phosphatidic acid (PA), and phosphatidylserine (PS), which carry a net negative charge at physiological pH [30].

Moreover, the significant shift in mobility observed at day 0 for curcumin-loaded nanoemulsions compared to their blank counterparts indicates that curcumin molecules are not solely sequestered within the oily core but are also intercalated within the interfacial phospholipid monolayer. The presence of curcumin at the interface likely induces a condensing effect, a phenomenon previously described in biophysical studies of curcumin-lipid bilayers [31,32,33].

Particle size distribution is expressed by the polydispersity index (PDI), a dimensionless parameter [34]. A PDI value below 0.3 typically indicates a narrow size distribution [35]. The PDI for all formulations ranged from 0.13 to 0.18 at Day 0, indicating that both empty and loaded nanoemulsions possessed a narrow, monodisperse size distribution.

Stability was monitored by quantifying changes in droplet size, PDI, and electrophoretic mobility over 30 days at 25 °C. As shown in Figure 3, these parameters remained relatively constant throughout the storage period. Furthermore, no macroscopic curcumin precipitation was observed. In general, emulsion stability is governed by the nature and concentration of the lipid phase, aqueous phase, and emulsifiers. Smaller droplet sizes typically confer higher kinetic stability due to increased Brownian motion (which opposes gravitational separation) and reduced creaming velocity, alongside enhanced steric stabilization [36].

Transmission electron microscopy (TEM) analysis (Figure 3C,D), performed using negative staining with phosphotungstic acid at 80 kV, revealed individual oil droplets that were uniformly spherical with smooth interfaces and well-defined boundaries. No evidence of coalescence or surface irregularities was observed. This indicates that the energy input during homogenization effectively disrupted oil aggregates to produce monodisperse droplets with high structural integrity. Such integrity is essential for ensuring consistent bioactive loading, minimizing Ostwald ripening, and achieving reproducible release kinetics in subsequent in vitro assays.

### 2.5. Cytotoxicity Analysis

The cytotoxic effect of empty and curcumin-loaded nanoemulsions on MCF-7 cancer cells was assessed using an impedance-based analysis system. To observe the influence of lipid composition, the effects of plant (rapeseed) and marine (salmon) nanoemulsions were compared. The samples were incubated with the cells during the exponential phase after 24 h of culture. The processing time was determined primarily by the rate of cell growth.

**Figure 4:**
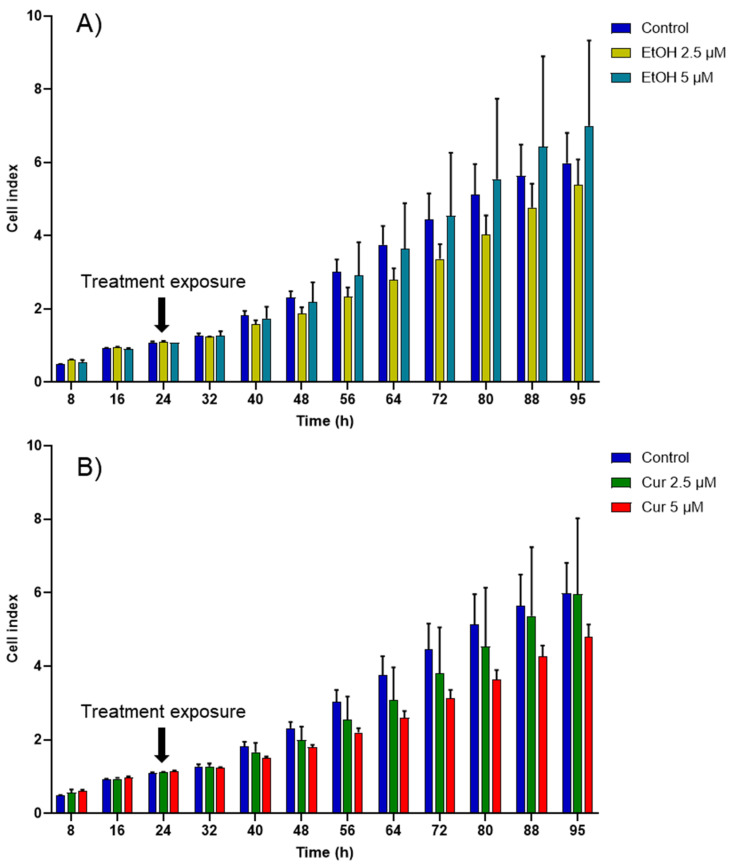
Cell index (CI) kinetics of the MCF-7 cells exposed to different concentrations of (**A**) ethanol (calculated corresponding to the used curcumin concentrations) and of (**B**) curcumin (Cur). CI was monitored during 71 h after compound exposure. Reported data are the means of three replicates (*n* = 3). All parametric data were analyzed using a two-way ANOVA followed by Dunnett’s test.

First, the cytotoxic effect of varying concentrations of non-encapsulated curcumin (2.5 and 5 µM) was investigated. Because curcumin was solubilized in ethanol, the potential cytotoxicity of ethanol was also evaluated. No significant cytotoxic effect was observed on MCF-7 cells exposed to the tested concentrations of ethanol or free curcumin (Figure 4). However, although statistically insignificant, the highest concentration of curcumin (5 µM) appeared to decrease the proliferation of MCF-7 the most.

Curcumin’s anti-proliferative activity has been previously attributed to induced apoptosis, involving proteins such as Bcl-2 and BAX [37,38]. Proteins of the BAX family are pro-apoptotic, while those of the Bcl-2 family are key regulators that inhibit apoptosis [39]. Specifically, BAX interacts with membrane pore proteins to increase cytochrome c release, whereas Bcl-2 inhibits this release [40]. The presence of curcumin results in an increase in BAX and a decrease in Bcl-2 at the cellular level [41]. Hence, the inhibition of anti-apoptotic proteins and the activation of pro-apoptotic proteins are two opposite mechanisms that confer anti-tumor activity to curcumin.

Other studies suggest that curcumin acts as an antioxidant and quenches reactive oxygen species (ROS) production at low concentrations, while inducing ROS production at high concentrations [42]. The antioxidant mechanism mediates NF-κB (nuclear factor-kappa B) suppressive effects, while the pro-oxidant mechanism mediates apoptotic effects [43]. Furthermore, curcumin uptake is reported to be higher in tumor cells than in normal cells, mainly due to differences in protein composition and membrane structure [44,45].

**Figure 5:**
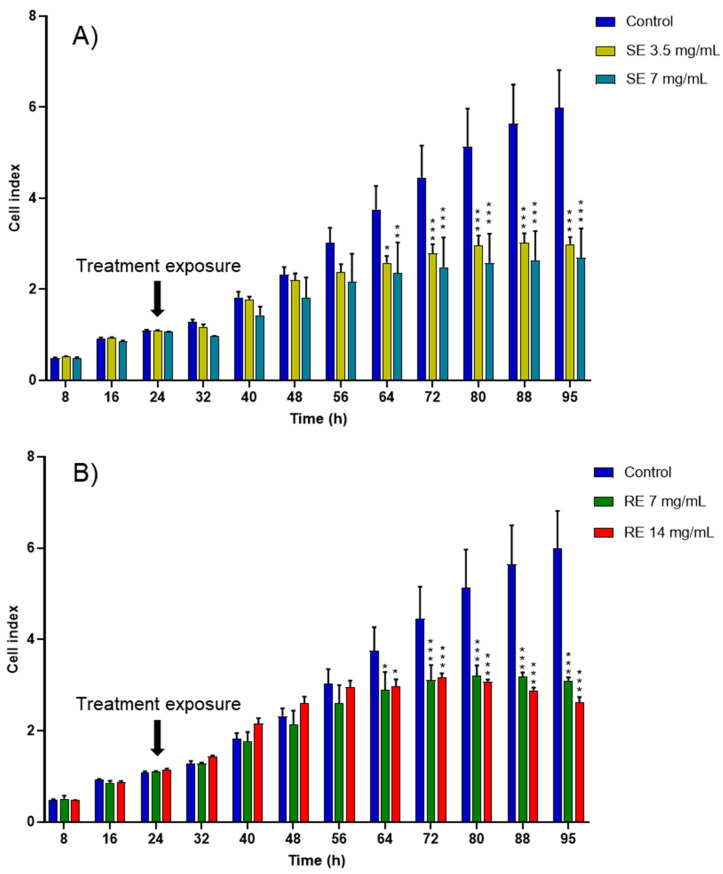
CI kinetics of the MCF-7 cells exposed to different concentrations of (**A**) salmon emulsions (SE) and (**B**) rapeseed emulsions (SE). CI was monitored during 71 h after liposomes exposure. Reported data are the means of three replicates (*n* = 3). Reported data are the means of three replicates. All parametric data were analyzed using a two-way ANOVA followed by Dunnett’s test. Significance was indicated as * (*p* < 0.05), ** (*p* < 0.01), and *** (*p* < 0.001).

Due to its extensive first-pass metabolism and poor aqueous solubility, curcumin cannot be used as an oral medication in its free form and without encapsulation in a delivery system, such as nanoemulsions. Nanoemulsions produced from salmon and rapeseed exhibited cytotoxicity toward cancer cells even without encapsulating curcumin (Figure 5), probably due to their fatty acid composition rich in n-3 PUFAs. The cytotoxic effect of n-3 PUFAs on cancer cells has been previously studied in vitro and has demonstrated a pro-oxidant potential [46,47,48].

Despite generally inconsistent epidemiological findings, animal and in vitro experiments have demonstrated that long-chain n-6 PUFAs possibly promote carcinogenesis, whereas n-3 fatty acids, particularly DHA and EPA, suppress the development of cancer [49]. Several studies have reported that a high ratio of n-6:n-3 fatty acids is associated with an increased risk of cancer, but the relationship with solely high n-6 intake is less clear [50]. The suggested biological mechanism for cancer prevention via long-chain n-3 fatty acid intake is the suppression of eicosanoid production from arachidonic acid [51,52].

**Figure 6:**
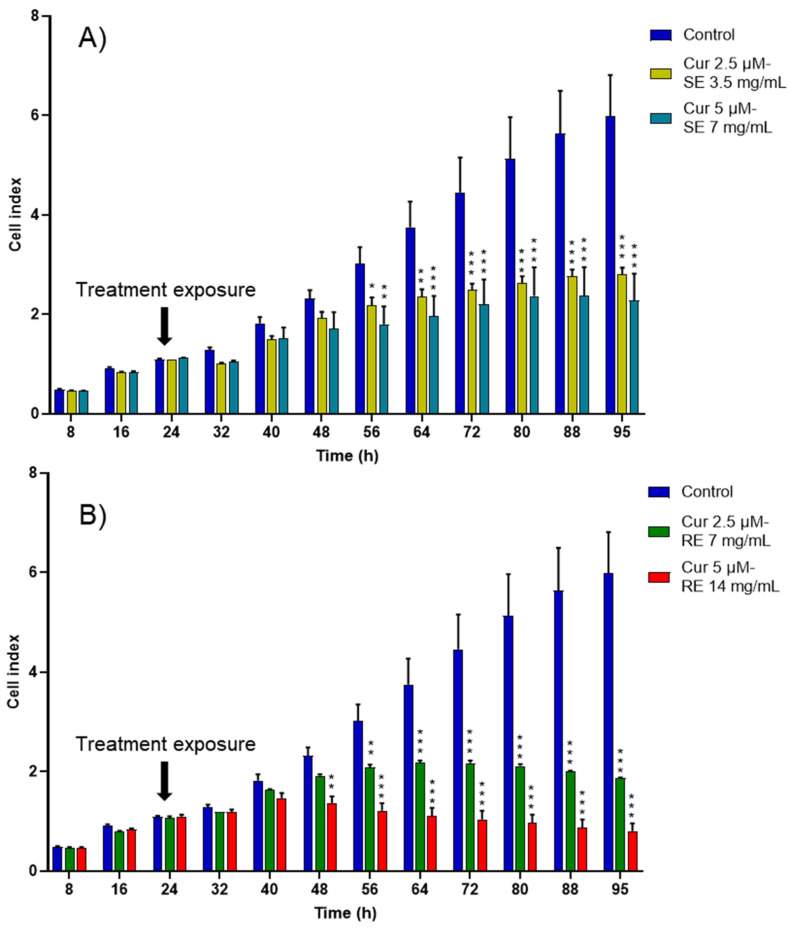
CI kinetics of the MCF-7 cells exposed to different concentrations of (**A**) salmon emulsions (SE) and (**B**) rapeseed emulsions (SE) loaded with curcumin (Cur). CI was monitored during 71 h after liposomes exposure. Reported data are the means of three replicates. Reported data are the means of three replicates (*n* = 3). All parametric data were analyzed using a two-way ANOVA followed by Dunnett’s test. Significance was indicated as * (*p* < 0.05), ** (*p* < 0.01), and *** (*p* < 0.001).

Notably, when curcumin was encapsulated inside rapeseed and salmon nanoemulsions, the cytotoxic effect towards cancer cells increased and appeared sooner than with empty formulations (Figure 6). A significant decrease in cancer cell proliferation appeared at 48 h for the high-concentration curcumin-loaded rapeseed nanoemulsions and at 56 h for the remaining curcumin-loaded formulations, whereas it only appeared after 64 h for the empty nanoemulsions.

Notably, while the high-concentration curcumin-loaded rapeseed emulsion decreased the proliferation of cancer cells the most, the concentration used was twice that of the salmon formulation due to the higher solubility of curcumin in the salmon nanoemulsion. Being able to encapsulate a high amount of curcumin even at low concentrations is a major advantage for salmon nanoemulsions, as it facilitates reaching a higher concentration of curcumin in the human plasma.

As shown in Table 2, the half inhibitory concentration (IC50) value of free curcumin was 12.1 ± 0.98 µM and the IC50 value of salmon emulsions was less than that of rapeseed emulsions. This superior cytotoxic effect might be due to the higher percentage of long chain fatty acids present in salmon oil and lecithin, especially EPA and DHA, and to the presence of astaxanthin, a red pigment of the xanthophyll (carotenoid) family, which is known for its antioxidant, anti-inflammatory, and anticancer properties [53,54,55]. Similar IC50 values were found for curcumin solution and curcumin-loaded nanoemulsions using leukemic and B16F10 cell lines [56]. Schultze et al. reported that MCF-7 cells treated with curcumin-loaded nanoemulsions showed the lowest inhibition rates at 24 h compared to cancer cells treated with curcumin-loaded lipid-core nanocapsules and curcumin DMSO-water solution [57]. Moreover, we can conclude from Table 2 that nanoemulsions and curcumin possessed a synergistic cytotoxic effect on MCF-7 breast cancer cells.

It is worth noting that while this study focused on the antiproliferative efficacy against MCF-7 breast cancer cells, the selectivity of the treatment toward malignant cells is a critical factor for clinical application. The formulations developed herein utilize food-grade lecithins (salmon and rapeseed) and curcumin, both of which are classified as GRAS (Generally Recognized As Safe) substances. Previous studies have extensively documented the selective cytotoxicity of curcumin, showing significant apoptotic effects on breast cancer cell lines while exhibiting minimal toxicity toward non-malignant cells at similar concentrations [58].

## 3. Materials and Methods

Salmon oil and salmon lecithin from Salmo salar produced without any organic solvent at low temperatures by enzymatic extraction [10]. Rapeseed lecithin and virgin rapeseed oil were purchased Solae Europe SA society (Geneva, Switzerland) and Huilerie d’Ormes (Ormes-et-Ville, France), respectively. Chloroform was obtained from VMR-Prolabo (Milan, Italy). Boron trifluoride (BF3)/methanol was obtained from Supelco Analytical (Bellefonte, PA, USA). Curcumin and acetonitrile were acquired from Sigma-Aldrich (Saint-Quentin-Fallavier, France). Hexane and methanol were obtained from Carlo-Erab (Val-de-Reuil, France). Cyclohexane, 2-propanol, toluene, methanol, hexane, and 1-propanol were purchased from Fisher (Illkirch, France). All organic solvents were analytical grade reagents.

### 3.1. Lipid Classes

Oils’ and lecithins’ lipidic classes were analyzed using an MK-5 TLC-FID Iatroscan (Iatron Laboratories Inc., Tokyo, Japan). Samples were first spotted on ten silica-coated quartz rods (Chromarod S-III). Rods were initially soaked in hexane/diethyl ether/formic acid (80:20:0.2, *v*:*v*:*v*) for 20 min, then dried for 1 min at 100 °C in an oven, before being scanned in the Iatroscan analyzer. The airflow rate was set at 2 L/min and the hydrogen flow rate at 160 mL/min. Polar lipids were quantified after a second migration by polar eluents of chloroform, methanol, and ammonia (65:35:5, *v*/*v*/*v*). Results represented the mean value of ten separate samples. To identify the sample component, the following standards, purchased from Sigma-Aldrich (Darmstadt, Germany), were used:

- Neutral lipids: cholesterol, tripalmitin, 1.2-dipalmitoyl-snglycerol, and 1-monostearoyl-rac-glycerol.
- Phospholipids: sphingomyelin, lyso-phosphatidylcholine, L-a-phosphatidylinositol, L-a-phosphatidyl-L-serine, 3 sn-phosphatidyleth-anolamine, and L-a-phosphatidylcholine.

ChromStar internal software (version 4.14) was used to record and integrate the peaks.

### 3.2. Fatty Acids Composition

FAMEs were synthesized via transmethylation as described by Morrison and Smith [59]. The transmethylation was accomplished using 1 mL of toluene and 1 mL of methanolic BF_3_ (14% *w*/*v*) at 100 °C. FAMEs extraction was achieved using cyclohexane, followed by a washing step with distilled water. The separation of FAMEs was carried out on a Shimadzu 2010 gas chromatography (Shimadzu, Noisiel, France) equipped with a flame-ionization detector (FID) and a SPTM2380 Supelco capillary column (60 m; 0.2 mm internal diameter × 0.25 μm film thickness (Bellefonte, PA, USA). The column temperature was initially set at 120 °C for 3 min, then this temperature was increased to 180 °C (2 °C/min) and held at 220 °C for 25 min. Injector and detector temperatures were set at 250 °C. Fatty acids were identified using standard PUFAs mixtures with C23 as internal standard, PUFA No.1 from a marine source, and PUFA No.2 from a vegetable source (Supelco, Sigma-Aldrich, Bellefonte, PA, USA). The identification of fatty acids was carried out by co-injection of standard compounds. The results were presented as the average of three repetitions.

### 3.3. Curcumin Solubility in Rapeseed and Salmon Oils

The solubility of curcumin was assessed at 37 °C by adding an excess amount of curcumin to 2 mL of oil while stirring for 72 h at 500 rpm in the dark, followed by centrifugation at 9000× *g* for 10 min at room temperature to separate the insoluble curcumin and recuperation of the supernatant that was then diluted with methanol. The absorbance of the supernatant was quantified at 425 nm by a spectrophotometer (Shimadzu UV-1605, Kyoto, Japan) and the insoluble amount of curcumin was calculated from a calibration curve. The results were the average of three repetitions.

### 3.4. Preparation of Differents Nanoemulsions Containing Curcumin

Marine (salmon) and vegetable (rapeseed) nanoemulsions were synthesized by adapting the protocol established by Belhaj et al. [13]. The formulations consisted of a 90% aqueous phase and a 10% lipid phase. The lipid fraction was composed of 7.66% oil and 2.33% lecithin, enriched with curcumin at its maximum solubility limits (0.33 mg/mL for salmon oil and 0.17 mg/mL for rapeseed oil). To prepare the samples, the components were blended at 55–60 °C and subjected to vigorous vortexing to achieve a homogenous pre-emulsion. The mixture underwent ultrasonication in an ice bath for 120 s at 40% amplitude, utilizing a pulsed cycle (1 s on, 1 s off). To produce nanoemulsions, 30 mL of the pre-nanoemulsion solution was processed for 5 cycles at 1500 bar with an Emulsiflex-C3p high-pressure homogenizer (Sodexim S.A, Muizon, France). Nanoemulsions were stored in the dark in an incubator at 25 °C.

### 3.5. The Size and Electrophoretic Mobility of Nanoemulsions

The nanoemulsions’ size, polydispersity index, and electrophoretic mobility were analyzed by Dynamic Light Scattering (DLS) method, using a Zetasizer Nano ZS apparatus (Malvern Instruments, Malvern, UK). The nanoemulsions were diluted with ultra-filtrate distilled water (1:400) and their scattering intensity was measured at a scattering angle of 173°, a refractive index of 1.471 and an absorbance of 0.01. The measurements were performed in triplicate at 25 °C.

### 3.6. The Stability of Nanoemulsion

The nanoemulsion formulations were stored for 30 days at 25 °C in a drying-cupboard and at days 0, 7, 14, 22 and 30 their size, polydispersity index, and electrophoretic mobility were analyzed again.

### 3.7. Transmission Electron Microscopy

To monitor the microstructure of nanoemulsions, transmission electron microscopy (TEM) was employed with a negative staining method. To reduce the concentration of nanoemulsions, they were diluted by 30-folds with distilled water, then mixed with an equal volume of a 2% ammonium molybdate solution and kept at ambient conditions for 3 min. One drop was pipetted on a Formvar/carbon-coated copper grid and left to dry at room temperature. Micrographs were obtained using a Philips CM20 (TEM) (Thermo Fisher Scientific, Hillsboro, OR, USA) operating at 200 kV and recorded using an Olympus TEM CCD camera (Olympus, Tokyo, Japan).

### 3.8. In Vitro Evaluation of the Anticancer Activity

The xCELLigence system (Roche Diagnostics GmbH, Mannheim, Germany) was employed to monitor the antiproliferative activity of curcumin-loaded nanoemulsions against MCF-7 cells. This system measures electrical impedance generated by cells attaching to microelectrodes embedded in the bottom of 96-well E-Plates™. This impedance is algorithmically converted into the CI, a metric that reflects changes in cell number, morphology, and viability [60].

Prior to treatment, MCF-7 cells (NCCS Pune) were cultured in RPMI 1640 medium lacking phenol red (Gibco, Waltham, MA, USA), supplemented with 10% FBS, 2 mM L-Glutamine, and 1% penicillin/streptomycin. The cultures were maintained at 37 °C with 5% CO2. Cells were seeded into E-Plates™ at a concentration of 10^4^ cells/well and incubated overnight to ensure attachment.

Experimental formulations, including empty and drug-loaded nanoemulsions as well as ethanol controls, were prepared by dilution in the growth medium. The system recorded CI values every 15 min to track proliferation dynamics over time. Experiments were performed in triplicate, and the data was analyzed to establish the IC_50_ values for each treatment.

### 3.9. Statistical Analysis

The data are presented as mean ± standard deviation. Statistical assessments were conducted using a two-way ANOVA with Dunnett’s post hoc test to compare multiple groups. Significant differences are denoted as * (*p* < 0.05), ** (*p* < 0.01), *** (*p* < 0.001).

## 4. Conclusions

This study aimed to develop and characterize curcumin-loaded nanoemulsions using salmon and rapeseed lipids, comparing their physicochemical properties and their ability to inhibit MCF-7 breast cancer cell proliferation. Our findings reveal that salmon oil demonstrated a markedly higher curcumin solubility capacity, with a maximum of 0.33 ± 0.07 mg/mL, which is nearly twice that observed in rapeseed oil (0.18 ± 0.05 mg/mL). This difference may be attributed to the distinct fatty acid composition and lipid class distribution of the marine-based oil compared to the plant-based source. Both nanoemulsion types exhibited excellent physical stability when stored for 30 days at 25 °C, evidenced by consistently maintained mean droplet diameters of approximately 170 nm for salmon and 220 nm for rapeseed formulations. Notably, curcumin incorporation resulted in a reduction in nanoemulsion droplet size relative to empty formulations, indicative of strong molecular interactions between curcumin and the lipid components, enhancing formulation compactness and stability.

The biological evaluation demonstrated that all nanoemulsion formulations significantly inhibited the proliferation of MCF-7 breast cancer cells compared to controls. This antiproliferative effect can be partially attributed to the inherent bioactivity of polyunsaturated fatty acids abundant in these systems, particularly EPA and DHA, which are known to exert anti-inflammatory and anticancer effects. Importantly, nanoemulsions loaded with curcumin produced a more rapid and pronounced reduction in cell proliferation compared to their empty counterparts, highlighting the synergistic potential of combining curcumin with lipid-based delivery vehicles. This enhanced efficacy may be linked to improved solubilization, protection from degradation, and facilitated cellular uptake of curcumin when encapsulated within the nanoemulsion matrix.

Taken together, these results establish that nanoemulsions formulated from naturally derived lipids extracted from salmon and rapeseed offer a viable and promising platform for the encapsulation and delivery of hydrophobic bioactive compounds such as curcumin. Their demonstrated physicochemical stability and potent antiproliferative effects against breast cancer cells underscore their potential utility in preventive and therapeutic applications. Further in vivo studies and mechanistic investigations are warranted to fully elucidate the pharmacokinetics, biodistribution, and anticancer mechanisms associated with these nanoemulsion systems.

## Figures and Tables

**Table 1:** Main fatty acid composition of different lecithins by gas chromatography (area %). SFA: saturated fatty acids; MUFA: monounsaturated fatty acids; EPA: eicosapentaenoic acid; DHA docosahexaenoic acid; PUFA: polyunsaturated fatty acids; (*n* = 3).

Fatty Acids	Salmon Oil		Rapeseed Oil		Salmon Lecithin		Rapeseed Lecithin	
	%	SD	%	SD	%	SD	%	SD
C14	3.87	0.03	-	-	1.60	0.01	-	-
C16	12.67	0.13	4.54	0.00	16.14	0.09	7.65	0.02
C18	3.21	0.02	1.45	0.00	4.68	0.02	1.46	0.01
C21	0.30	0.30	-	-	1.93	0.02	-	-
C22	-	-	-	-	0.78	0.29	0.29	0.29
C23	-	-	-	-	1.18	0.07	-	-
SFA	20.05	-	5.99	-	26.31	-	9.4	-
C16:1	4.29	0.02	0.25	0.00	1.54	0.06	0.29	0.00
C17:1	0.39	0.01	-	-	1.20	0.01	-	-
C18:1n9	36.36	0.23	63.02	0.06	19.96	0.30	53.75	0.23
C20:1n11	5.17	0.12	1.21	0.01	0.42	0.10	0.68	0.01
MUFA	46.21	-	64.48	-	23.12	-	54.72	-
C18:2n6	11.46	0.18	18.81	0.07	5.81	0.07	27.95	0.06
C18:3n3	4.24	0.08	9.22	0.03	2.70	0.02	6.54	0.09
C20:2n6	1.52	0.02	0.25	0.02	0.29	0.03	0.17	0.02
C20:3n6	0.27	0.00	-	-	0.30	0.02	-	-
C20:3n3	0.40	0.00	-	-	0.31	0.04	-	-
C20:4n6	0.50	0.72	-	-	2.32	0.10	-	-
C20:5n3(EPA)	4.94	0.02	-	-	9.40	0.06	-	-
C22:4n6	0.52	0.01	-	-	1.68	0.03	-	-
C22:5n3	2.46	0.05	-	-	3.22	0.06	-	-
C22:6n3(DHA)	6.43	0.04	-	-	23.41	0.29	-	-
PUFA	32.74	-	28.28	-	49.13	-	34.66	-
n-3/n-6	1.28	-	0.51	-	3.75	-	0.23	-
DHA/EPA	1.30	-	-	-	2.49	-	-	-

**Table 2:** The half inhibition concentration (IC50) values of curcumin (Cur), salmon emulsions (SE), and rapeseed emulsions (RE) on MCF-7 cells (*n* = 3).

	Cur	SE	RE	Cur-SE	Cur-RE
IC50	12.1 ± 0.98 µM	5.66 ± 0.51 mg/mL	9.37 ± 0.55 mg/mL	3.96 ± 0.32 mg/mL SE 2.83 ± 0.23 µM Cur	4.37 ± 0.12 mg/mL RE 1.56 ± 0.04 µM Cur

## Data Availability

The original contributions presented in this study are included in the article. Further inquiries can be directed to the corresponding author.
